# Research progress of extracellular vesicles in the pathogenesis of type IIIA chronic prostatitis

**DOI:** 10.3389/fimmu.2025.1496055

**Published:** 2025-02-17

**Authors:** Le Cheng, Peiyue Luo, Wei Li, Qi Chen, Lifeng Gan, Fangtao Zhang, Liying Zheng, Biao Qian

**Affiliations:** ^1^ The First Clinical College, Gannan Medical University, Ganzhou, Jiangxi, China; ^2^ Department of Urology, The First Affiliated Hospital of Gannan Medical University, Ganzhou, Jiangxi, China; ^3^ Key Laboratory of Urology and Andrology of Ganzhou, Institute of Urology, First Affiliated Hospital of Gannan Medical University, Ganzhou, Jiangxi, China; ^4^ Department of Graduate, The First Affiliated Hospital of Gannan Medical University, Ganzhou, Jiangxi, China

**Keywords:** extracellular vesicles, type IIIA chronic prostatitis, interactive functional modulation, application potential, molecular mechanism

## Abstract

Chronic prostatitis is a prevalent urological condition that significantly impacts patients’ quality of life. Advances in the study of Extracellular Vesicles (EV) have revealed their close involvement in the pathogenesis of prostatitis. This paper reviews the progress in understanding the role of EV in the pathogenesis of chronic prostatitis type IIIA, particularly their involvement in inflammatory responses, cell signaling, and interactions with immune cells. Additionally, it explores the potential applications of EV as drug delivery vehicles, including the targeted delivery of anti-inflammatory agents and immunomodulators, and highlights the challenges associated with developing exosome-based therapeutic strategies. In-depth research on EV holds promise for offering new insights into the diagnosis and treatment of inflammatory diseases.

## 1 Introduction

The pathogenesis of chronic prostatitis is complex and involves various cellular and molecular pathways (1, 2). According to the classification by the National Institutes of Health (NIH), Type IIIA chronic prostatitis is relatively common among chronic prostatitis cases. Currently it is believed that abnormalities in immune responses, infectious factors, neuroendocrine dysregulation, and oxidative stress may all contribute to the development and progression of this disease. In recent years, with the research on extracellular vesicles, their role as an important medium of intercellular communication in chronic prostatitis is often mentioned. Extracellular Vesicles (EV) are small vesicles encased by the cell membrane, containing bioactive molecules such as proteins, lipids, mRNA, and miRNA. They can transmit information between cells and regulate the functions of target cells (3–5). During the pathological process of prostatitis, EV may play a crucial role by mediating the activation of inflammatory cells, promoting the release of inflammatory factors, and modulating interactions among immune cells. This review will summarize recent advances in exosome research related to type IIIA chronic prostatitis, explore their role in disease mechanisms, and discuss their potential as therapeutic targets.

In the investigation of the pathogenesis of chronic prostatitis type IIIA, it is essential to address the role of EV, a critical biomolecule. EV are small vesicles secreted by cells, characterized by a unique lipid bilayer structure and containing a rich array of biomolecules such as proteins, RNA (6), and DNA (7). These molecules endow EV with a crucial role in intercellular communication. Exosomes facilitate intercellular communication and signal transduction by carrying and transferring genetic information and proteins (8, 9).

EV exhibit biological characteristics that confer high stability, biocompatibility, and specific surface markers, such as CD63 and CD81 (8, 10). These features suggest that EV hold significant potential for applications in the biomedical field (11). They may play a crucial role in the pathogenesis of chronic prostatitis type IIIA (12, 13). As mediators of intercellular communication, EV might be involved in the inflammatory response and pathological processes associated with chronic prostatitis type IIIA (14). By transmitting inflammatory signals and modulating immune responses, EV could potentially promote disease progression and exacerbation.

Therefore, we can consider utilizing the molecular information carried by exosomes to aid in the diagnosis and treatment of Type IIIA chronic prostatitis (15, 16). By detecting exosome biomarkers in prostate secretions, early diagnosis of type IIIA chronic prostatitis can be achieved, and targeted therapies can be developed (17). Furthermore, studying the role of EV in the pathogenesis of type IIIA chronic prostatitis contributes to a deeper understanding of the disease and offers new insights and methods for its diagnosis (18), treatment (19), and prevention (20).

To further investigate the role of EV in type IIIA chronic prostatitis, various experimental methods and techniques can be employed (2). For instance, molecular biology techniques can be used to isolate and purify EV, followed by the analysis of the molecular information they carry (21). Additionally, animal models and cell experiments can simulate the pathogenesis of type IIIA chronic prostatitis and allow for the observation of exosome effects (22).

In summary, Type IIIA chronic prostatitis is a prevalent urogenital disorder characterized by a complex and heterogeneous etiology. EV as pivotal mediators of intercellular communication, are likely to play a significant role in the pathogenesis of this condition. Research into the function of EVs in Type IIIA chronic prostatitis may yield novel insights and therapeutic strategies, potentially enhancing diagnostic accuracy, treatment efficacy, and preventative measures, thereby improving patient outcomes and quality of life.

## 2 Relationship between extracellular vesicles and type IIIA chronic prostatitis

EV, as nanoscale vesicles, are crucial mediators of intercellular communication and material transport (1). Recent advancements in exosome research have increasingly highlighted their significant role in prostate tissue. This paper will provide a detailed examination of the sources and distribution of EV within prostate tissue, as well as their interactions with prostate cells. Furthermore, it will explore the potential role of EV in the development and progression of prostatitis.

### 2.1 Origin and distribution of extracellular vesicles

The presence of EV in prostate tissue is both extensive and intricate, playing a crucial role in the exchange of information and transport of substances between prostate cells (23). These minute vesicular structures are not only found in prostate epithelial cells but are also widely distributed among various cell types, such as stromal cells and infiltrating inflammatory cells (24). Under physiological and pathological conditions, these cells release EV into bodily fluids, including prostatic secretions, through a series of complex and subtle mechanisms, participating in various physiological and pathological processes within the prostate tissue (25, 26). Prostatic epithelial cells are among the most critical cell types within prostatic tissue, playing an essential role in the male reproductive and urinary systems through the secretion of prostatic fluid (27). Ion and release of EV by these epithelial cells is a continuous and dynamic process. The biogenesis and secretion of EV in epithelial cells represent a continuous and dynamic process. These vesicles, rich in bioactive substances, play a pivotal role in intercellular communication, modulation of gene expression, and immune responses (8, 10, 28).

In addition to epithelial cells, stromal cells in prostate tissue are also significant sources of EV. These mesenchymal cells primarily include smooth muscle cells and fibroblasts, which play a crucial role in providing structural support and regulating function within prostate tissue (11). The EV released by these cells may participate in processes such as angiogenesis, inflammatory responses, and cell proliferation within the prostate. Furthermore, infiltrating inflammatory cells in prostate tissue are also key contributors to exosome release (13). Under pathological conditions like inflammation, these inflammatory cells release a substantial amount of EV containing numerous inflammatory mediators and signaling molecules (12). These EV can impact the microenvironment of the prostate, thereby affecting its function and structure.

EV are ubiquitously present in bodily fluids, including those found in prostatic fluid. These exosomes not only engage in signal transduction, material transport within prostatic cells (14), and various physiological and pathological processes (29). Therefore, a thorough investigation into the origins, release mechanisms, and functional roles of exosomes within prostatic tissue is essential. Such in-depth research on these vesicles can enhance our understanding of the structure and function of prostatic tissue, and offer new perspectives and methodologies for the diagnosis and treatment of prostatic diseases. Additionally, it is of significant importance for elucidating the pathogenesis of prostatic diseases and for the development of novel therapeutic strategies.

### 2.2 Interaction between extracellular vesicles and prostate cells

EV as one of the complex and subtle intercellular communication mechanisms in biological systems, play an indispensable role by binding closely to receptors on the surface of prostate cells (30). This unique communication mechanism not only facilitates signal transmission between cells but also promotes material exchange, thereby providing a solid foundation for maintaining the internal environment of the organism.

In intercellular signaling, EV function as messengers (31). They transport a variety of bioactive molecules, such as proteins, nucleic acids, and lipids, which have a strong ability to regulate cellular functions (32). Upon binding to receptors on prostate cell surfaces, these active molecules can be released and directly influence the physiological functions of prostate cells (33). For instance, certain protein molecules can modulate the proliferation, differentiation, and apoptosis of prostate cells, while nucleic acids may participate in gene expression regulation, thereby affecting the metabolic activity of prostate cells (34, 35). Conversely, prostate cells can internalize EV through endocytosis, acquiring exogenous substances. This internalization process not only provides essential nutrients and energy sources but also enables cells to regulate their metabolism and signaling pathways (36). Through this mechanism, prostate cells can rapidly respond to changes in the external environment and maintain internal homeostasis.

It is noteworthy that the interaction between EV and prostate cells is not unidirectional. Instead, it is a dynamic, bidirectional process involving the synergistic action of various intercellular signaling and substance exchange pathways (37). This synergy enables EV to play a crucial role in the physiological and pathological processes of prostate cells. Furthermore, as research progresses, scientists have discovered that EV may also be closely related to the pathogenesis of diseases such as prostate cancer. For instance, some studies indicate that prostate cancer cells might influence the growth and differentiation of surrounding normal cells by releasing specific EV, thereby promoting tumor development (38). Therefore, a deeper exploration of the mechanisms underlying exosome-prostate cell interactions not only aids in understanding the complexity and diversity of intercellular communication but may also provide new insights and directions for the prevention and treatment of diseases like cancer.

EV facilitate intercellular signal transduction and substance exchange by binding to receptors on the surface of prostate cells. This intricate interaction mechanism not only reveals the mysteries of cell-to-cell communication but also provides valuable insights for further exploration of the secrets of life sciences (39).

### 2.3 The role of extracellular vesicles in type IIIA chronic prostatitis

Type IIIA chronic prostatitis is a complex and common male urological disorder, with its pathogenesis involving multiple biological processes and pathological mechanisms. In recent years, as research into intercellular communication mechanisms has advanced, EV have emerged as crucial mediators of cell-to-cell communication. They may play a vital role in the pathogenesis of Type IIIA chronic prostatitis.

In the pathogenesis of chronic prostatitis type IIIA, EV may be involved in various mechanisms (40). On one hand, EV may contribute to the inflammatory response in prostate tissue by transporting inflammation-related molecules such as inflammatory mediators and chemokines (41). These molecules can be delivered to target cells via EV, activating inflammatory signaling pathways, promoting the infiltration of inflammatory cells, and the release of inflammatory mediators (42). This process may lead to sustained inflammation within the prostate tissue, exacerbating tissue damage and functional impairment (43). On the other hand, EV may also play a role in the remodeling and repair processes of prostate tissue, influencing the pathogenesis of type IIIA chronic prostatitis (44). After prostate tissue damage, a series of complex biological processes are required for repair and remodeling. EV can carry growth factors and extracellular matrix components, which are crucial for the proliferation, differentiation, and structural reconstruction of prostate cells (45). By delivering these bioactive molecules, EV can facilitate the repair and remodeling of prostate tissue, thereby potentially mitigating disease progression.

In addition to the two primary aspects mentioned, EV may also be associated with other pathological mechanisms of type IIIA chronic prostatitis (46). For instance, EV might be involved in regulating the immune system, thereby affecting the distribution and function of immune cells within prostate tissue (47). Moreover, EV could have a profound impact on prostate tissue health by influencing biological processes such as apoptosis and autophagy.

## 3 Recent advances in the role of extracellular vesicles in the pathogenesis of type IIIA chronic prostatitis

### 3.1 Role of extracellular vesicles in inflammation regulation

**Figure 1:**
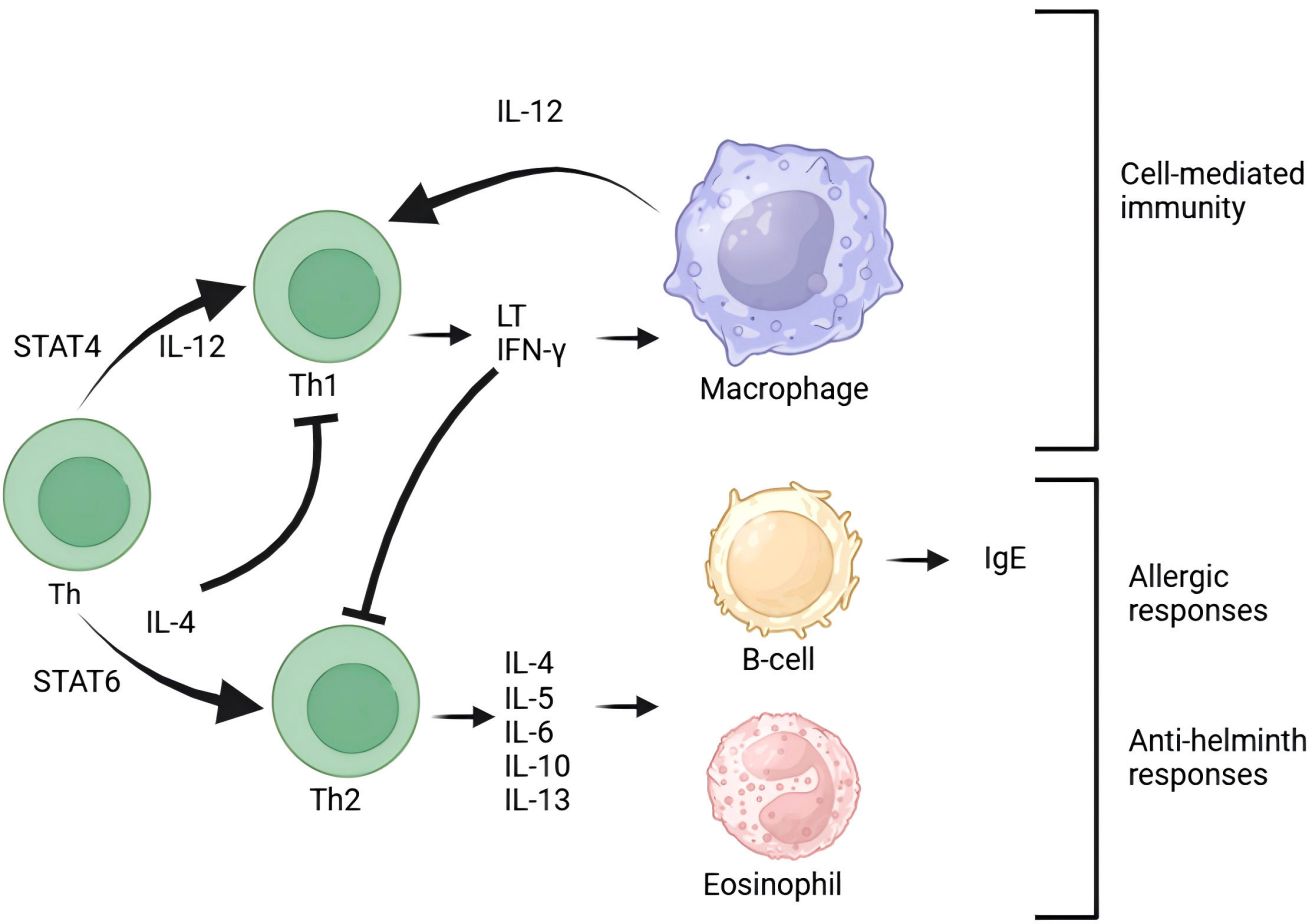
T cells are central to the immune response and maintain close interactions with other immune cells. B cells are responsible for antibody production and work in conjunction with T cells to contribute to immune defense. Macrophages, a type of white blood cell, are tasked with phagocytosing and digesting foreign particles, thereby participating in immune responses. Additionally, cytokines such as IFN-γ and IL-12 play crucial roles in activating immune cells. The presence of eosinophils indicates that the immune system is involved in allergic reactions. Exosomes influence the immune response process by modulating various signaling pathways.

In the pathogenesis of IIIA chronic prostatitis, EV play a crucial role in inflammation regulation. As significant carriers of intercellular communication, EV can transport and convey various inflammation-related molecules, such as inflammatory mediators (48), chemokines (49), and immunoregulatory molecules (50), thereby modulating the inflammatory response (**Figure 1**).

EV regulate the inflammatory response in chronic prostatitis type IIIA through various signaling pathways. On one hand, EV can carry and deliver specific signaling molecules, such as protein kinases (51) and transcription factors (52). These molecules can activate or inhibit signaling pathways within prostate cells, thereby modulating the expression of inflammation-related genes and the intensity of the inflammatory response (53). On the other hand, EV can bind to receptors on the surface of prostate cells, and through processes like membrane fusion or endocytosis, transfer signaling molecules into the cell, thereby triggering or inhibiting intracellular signaling pathways and influencing the progression of inflammation.

EV can participate in the transmission of inflammatory signals in prostate tissue. In the pathogenesis of chronic prostatitis type IIIA, cells within the prostate tissue release EV in response to various stimuli. These EV carry inflammatory molecular information and convey inflammatory signals to neighboring cells through intercellular communication, thereby triggering or exacerbating the inflammatory response (54).

EV can regulate the proliferation and apoptosis of prostate cells. In inflammatory environments, the proliferation and apoptosis of prostate cells often become dysregulated. EV can influence these processes by delivering specific molecular information, thereby affecting the onset and progression of prostatitis.

In addition, EV are involved in the regulation of the immune system. The onset of chronic prostatitis type IIIA is often accompanied by abnormal activation of the immune system (55). EV can carry immune regulatory molecules, modulating the immune response by affecting the activity and function of immune cells, thereby significantly influencing the onset and progression of prostatitis.

Recent advances in exosome research have increasingly demonstrated that EV play a crucial role in the pathogenesis of Type IIIA chronic prostatitis (56). Further investigation into the biological characteristics of EV and their mechanisms of action in prostatitis is expected to provide new insights and methods for the diagnosis, treatment, and prevention of the disease.

In the future, advanced molecular biology techniques and experimental methods can be employed for a more in-depth study of EV. For instance, high-throughput sequencing technology can be used to sequence and analyze RNA within EV to elucidate their regulatory roles in prostatitis (57). Additionally, animal models and cell experiments can simulate the pathogenesis of prostatitis to observe the mechanisms of exosome action. These studies will contribute to a more comprehensive understanding of the role of EV in the pathogenesis of chronic prostatitis type IIIA, providing new insights and methods for the prevention and treatment of the disease.

### 3.2 Relationship between extracellular vesicles and proliferation and apoptosis of prostate cells

EV as crucial mediators of intercellular communication, play an indispensable role in the proliferation and apoptosis of prostate cells. By transferring specific molecular information, they precisely regulate cellular growth, differentiation, and death, thereby having a profound impact on the pathogenesis and progression of Type IIIA chronic prostatitis.

EV play a role in regulating prostate cell proliferation. Under normal physiological conditions, the proliferation and differentiation of prostate cells are tightly regulated to maintain tissue homeostasis (58). However, during episodes of chronic prostatitis type IIIA, this homeostasis is often disrupted. EV can carry a variety of growth factors, hormones, and other signaling molecules, and modulate the proliferation rate and differentiation direction of prostate cells by influencing intracellular signaling pathways (59). Abnormal expression of certain molecules in EV may lead to excessive proliferation or abnormal differentiation of prostate cells, thereby exacerbating inflammation and tissue damage.

**Figure 2:**
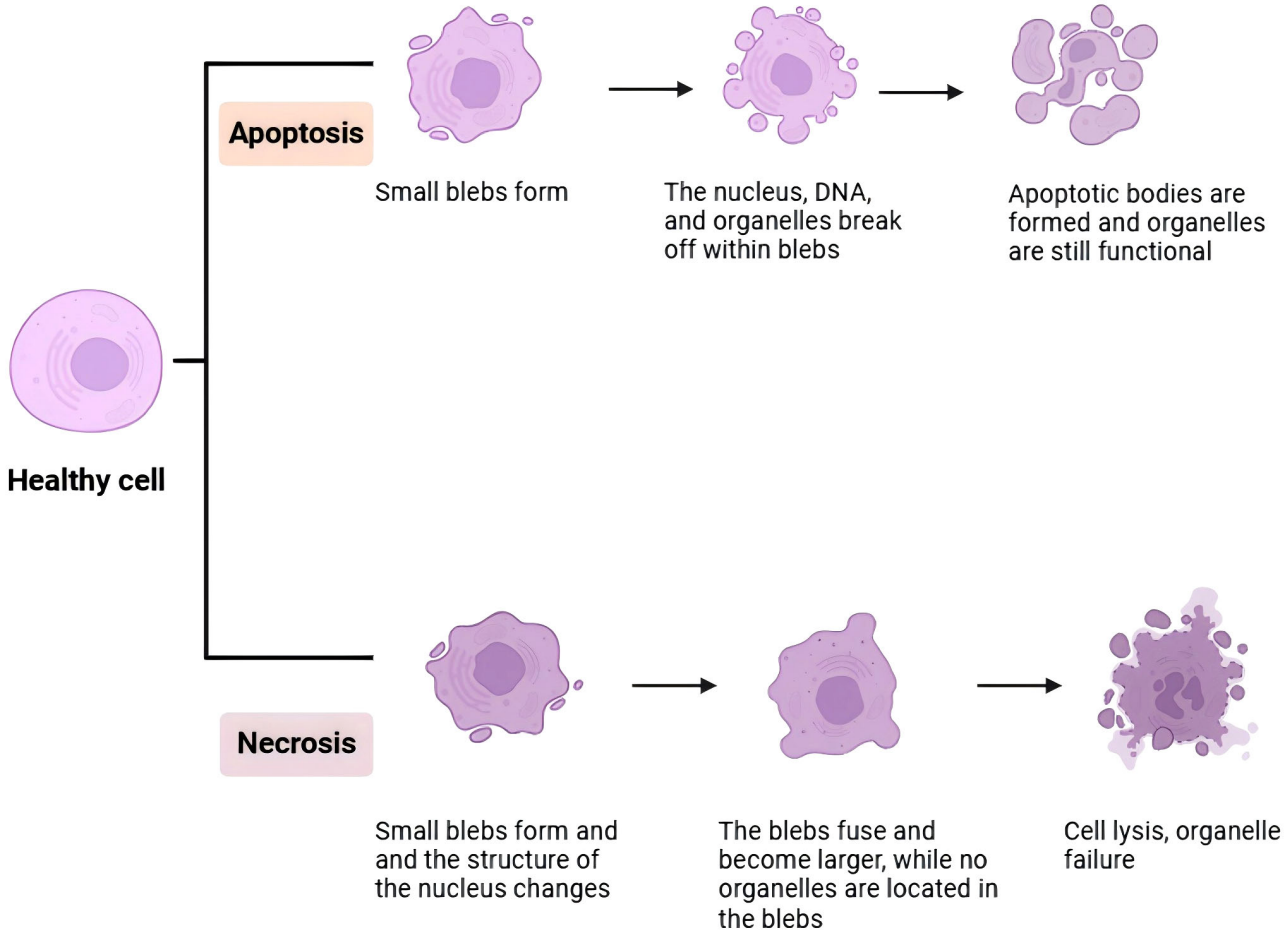
In this image, a detailed diagram of the immune response against colon cancer is presented. It illustrates the interaction between tumor cells, immune cells, and various signaling molecules. The tumor cells, represented in red, are targeted by immune cells such as T cells depicted in blue and macrophages depicted in green. The diagram also emphasizes the involvement of cytokines like IL-10 and TNF-α, which play pivotal roles in regulating the immune response. The overall flowchart provides a comprehensive overview of the complex interactions between tumor cells and immune cells, ultimately leading to the suppression of tumor growth.

EV play a crucial role in the process of apoptosis in prostate cells (**Figure 2**). Apoptosis is a programmed cell death process that is vital for maintaining tissue homeostasis and preventing disease onset (60). EV can trigger apoptosis in prostate cells by transferring apoptosis-related molecules, such as apoptosis-inducing factors and cytochrome C. In chronic prostatitis of type IIIA, EV may modulate the expression of apoptosis-related molecules, influencing the rate and extent of prostate cell apoptosis, thereby affecting disease progression and prognosis.

In the detailed study of the relationship between EV and prostate cell proliferation and apoptosis, signaling pathways have emerged as a crucial research focus. These pathways are essential for the transmission of both intracellular and extracellular signals, ensuring that cells appropriately respond to various internal and external stimuli. In the pathogenesis of type IIIA chronic prostatitis, EV regulate prostate cell proliferation and apoptosis by influencing specific signaling pathways, thereby contributing to disease progression (61). Many growth factors, hormones, and cytokines activate downstream signaling cascades by binding to their receptors, ultimately affecting gene transcription and expression in the cell nucleus, which in turn regulates cell proliferation (62). EV can carry these growth factors or hormones and deliver them to prostate cells, modulating the rate of cell proliferation by activating or inhibiting these signaling pathways. For instance, molecules carried by EV may activate proliferative signaling pathways such as PI3K/Akt or MAPK, leading to excessive proliferation of prostate cells; other molecules might inhibit these pathways, thereby suppressing proliferation.Apoptosis is a complex process of cell death involving the coordinated action of multiple signaling pathways (63). EV can trigger or inhibit prostate cell apoptosis by transferring apoptosis-related molecules. For example, certain molecules carried by EV may activate caspase family proteins, initiating a cascade of apoptotic events; other molecules may promote apoptosis by inhibiting the expression of anti-apoptotic proteins (64). The regulation of these apoptosis-related signaling pathways is crucial for maintaining prostate tissue homeostasis and preventing disease progression.

Moreover, EV may be closely associated with the autophagic process in prostate cells. Autophagy is the process by which cells digest and recycle internal materials, a mechanism crucial for maintaining cellular homeostasis and function. Research indicates that EV can carry autophagy-related proteins and signaling molecules (65), thereby participating in the regulation of autophagy in prostate cells. In chronic prostatitis type IIIA, EV may modulate autophagy levels in prostate cells by influencing the expression and activity of autophagy-related molecules, thereby affecting cellular survival and death.

In summary, EV play a complex and precise regulatory role in the proliferation and apoptosis of prostate cells (66). They affect the growth, differentiation, and death processes of prostate cells by transmitting specific molecular information, thereby participating in the pathogenesis and progression of type IIIA chronic prostatitis. Future research should delve deeper into the biological characteristics and functional mechanisms of EV to provide new insights and methods for disease prevention and treatment.

### 3.3 Interactions between extracellular vesicles and the prostate immune system

The interaction between EV and the prostate immune system plays a significant role in the pathogenesis of chronic prostatitis type IIIA. As a complex organ, the balance and stability of the prostate immune system are crucial for maintaining normal physiological functions (67). EV, serving as critical mediators in intercellular communication, are capable of modulating the activity and functionality of prostate immune cells, which in turn can influence the intensity and progression of the inflammatory response.

**Figure 3:**
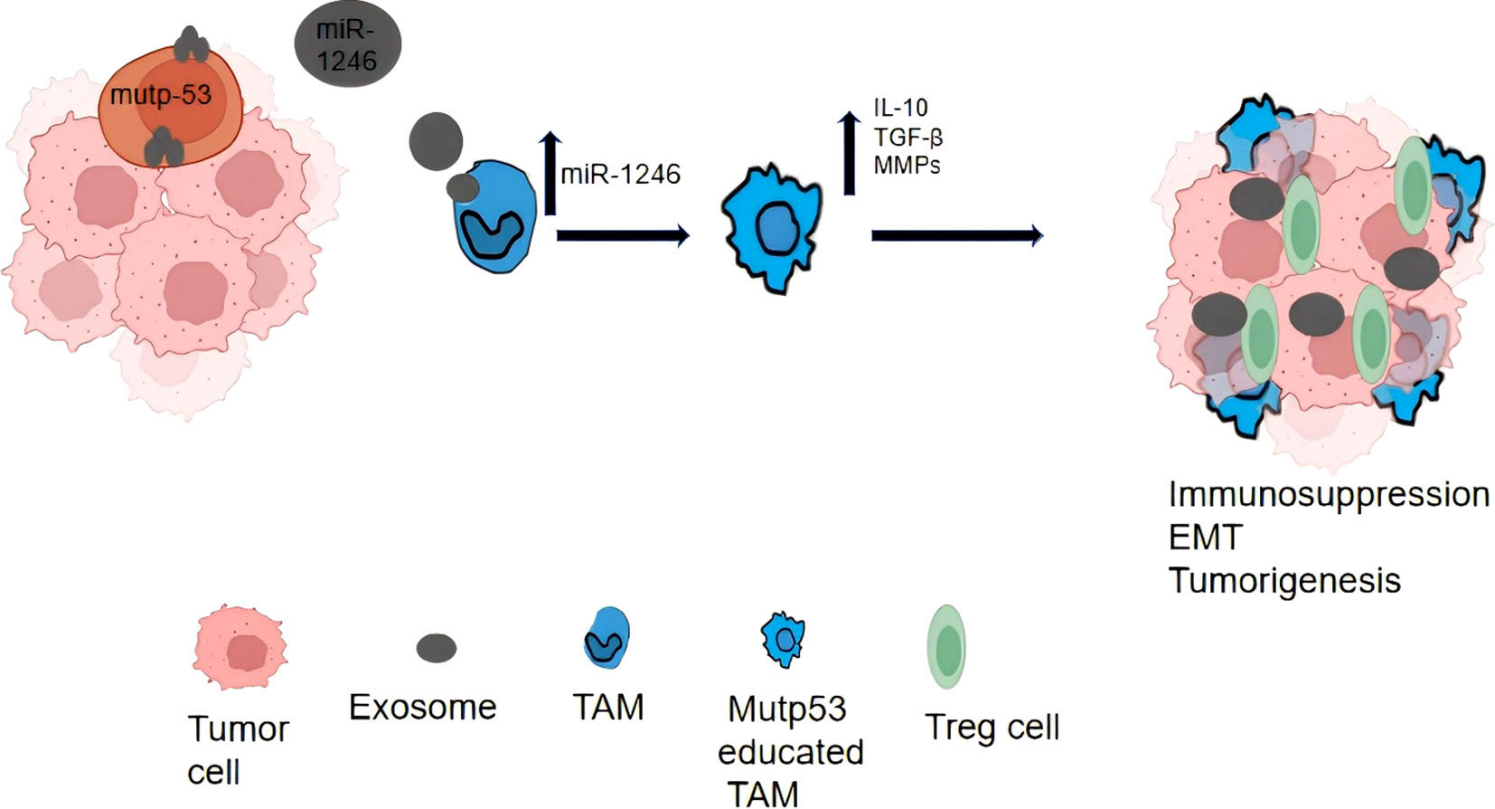
If a cell undergoes apoptosis, small, balloon-like structures will form, within which the nucleus, DNA, and organelles fragment; the apoptotic bodies that result still retain functionality. In contrast, during necrosis, these balloon-like structures merge and enlarge, while the nucleus and organelles remain within the structures. Eventually, the cell dissolves, leading to a loss of organelle function.

EV play a role in the activation and differentiation of prostate immune cells. During an acute episode of chronic prostatitis type IIIA, immune cells in the prostate tissue are stimulated and release EV (68). These EV carry immunomodulatory molecules, such as chemokines and immunosuppressive factors, which can influence the activation and differentiation of immune cells (69). By modulating the activity and function of immune cells, EV can regulate the response of the prostate immune system, thereby affecting the onset and progression of prostatitis (**Figure 3**).

EV can influence the migration and infiltration of immune cells in the prostate. In an inflammatory environment, immune cells in the prostate migrate to the sites of inflammation and participate in the inflammatory response. EV regulate the extent and severity of the inflammatory response by modulating the migration and infiltration of immune cells (70). Abnormal expression of certain molecules within EV may lead to excessive infiltration of immune cells or hinder their migration, thereby exacerbating the inflammatory response or affecting its resolution.

EV can also play a role in the apoptosis and autophagy of prostate immune cells. Apoptosis and autophagy are crucial mechanisms for maintaining the balance and function of immune cells within the body (71). EV can influence the survival and death of immune cells by transferring molecules related to apoptosis and autophagy. In type IIIA chronic prostatitis, EV may impact immune system responses and disease progression by modulating the levels of apoptosis and autophagy in immune cells.

In the investigation of exosome interactions with the prostate immune system, signaling pathways play a crucial role. These pathways act as bridges for information exchange both within and outside the cell, regulating various biological processes including the activation, differentiation, migration, and apoptosis of immune cells (72, 73). EV participate in the regulation of signaling pathways in prostate immune cells by carrying and transmitting specific signaling molecules, thereby influencing immune system function and response. When EV bind to receptors on the surface of immune cells, they can trigger a cascade of signals that activate or inhibit specific pathways (74). These pathways, in turn, regulate gene expression, cell proliferation, differentiation, and the execution of immune cell functions. In type IIIA chronic prostatitis, EV may modulate signaling pathways within immune cells, affecting the intensity and duration of immune responses and contributing to disease onset and progression (75). For instance, TRPM8 RNA is secreted by both normal and prostate cancer cells via EV without inducing cell damage. Upon internalization of EV, TRPM8 mRNA binds to TLR3 within endosomes, thereby promoting the activation of the NF-kB/IRF3 pathway and the release of pro-inflammatory signals. The delivery of TRPM8 mRNA via EV activates TLR3, which triggers sterile inflammation in prostate epithelial cells and enhances the inhibitory effect of NK cells on tumor growth (76). Inflammation is a crucial response of the immune system to injury or infection, but excessive inflammation can lead to tissue damage and disease progression. EV can modulate inflammation-related signaling pathways in prostate immune cells by carrying anti-inflammatory or pro-inflammatory molecules, thereby balancing immune responses and preventing excessive inflammation (77). Increased expression of anti-inflammatory molecules in EV may inhibit excessive activation of immune cells and alleviate inflammation, whereas increased expression of pro-inflammatory molecules may enhance immune responses and promote inflammation resolution.

EV regulate immune system functions and responses by modulating signaling pathways within prostatic immune cells. In the pathogenesis of chronic prostatitis type IIIA, EV may influence the degree and progression of inflammation by affecting the activation, differentiation, migration, apoptosis, and autophagy of immune cells. This, in turn, can have profound effects on the disease’s pathogenesis and progression. Experiments have revealed that the combination of PD-1 inhibitors with docetaxel exerts a synergistic effect on mouse prostate cancer, inhibiting tumor growth, enhancing survival rates, and reducing adverse reactions. This combination also increases tumor-infiltrating CD4+ and CD8+ T cells, particularly when used in conjunction with low-dose docetaxel, which is associated with the PI3K/AKT/NFKB-P65/PD-L1 signaling pathway (78). Studies indicate that numerous cytokines in the tumor microenvironment and tumor-derived EV can induce the expression of PD-L1, facilitating tumor immune evasion (79). Consequently, targeting the PD-1/PD-L1 axis with immune checkpoint inhibitors (ICIs) has become one of the primary strategies in cancer immunotherapy to reverse immunosuppression and restore the immune system’s antitumor activity (80). Future research should further investigate the mechanisms underlying the interactions between EV and the prostatic immune system, potentially offering new approaches and strategies for disease prevention and treatment.

## 4 Exploration of extracellular vesicles as therapeutic targets for type IIIA chronic prostatitis

EV as an emerging drug delivery system, have garnered significant attention in the biomedical field due to their unique advantages. As the primary form of EV, EV originate from cells and possess the ability to fuse with host cell membranes, enabling the direct delivery of drugs to target cells. This characteristic endows EV with high targeting capability in drug delivery. For example, by precisely modulating the source and function of EV, researchers have successfully directed anti-inflammatory drugs to sites of inflammation (81). This targeted approach not only enhances therapeutic efficacy but also significantly reduces the incidence of side effects (62). Compared to traditional drug delivery methods, EV can more accurately reach the lesion sites, achieve precise drug release, and thereby improve overall treatment effectiveness.

Additionally, EV exhibit high biocompatibility and stability. These nanoscale vesicles are naturally secreted by cells and can persist in the body, effectively evading immune system surveillance. This characteristic renders EV as relatively safe drug carriers (82). Furthermore, the membrane material of EV originates from the cell membrane, ensuring good compatibility with human tissues and further reducing the risk of immune responses. Notably, EV also possess a strong drug-loading capacity. They can carry a variety of biomolecules, such as proteins, RNA, and DNA, enabling multi-targeted therapies (83, 84). This multi-drug combination strategy enhances therapeutic efficacy while simultaneously reducing the dosage and potential side effects of individual drugs.

EV exhibit substantial potential in the dissemination of immunomodulators. By regulating immune-modulating factors such as miRNAs and lncRNAs within EV, researchers can achieve precise modulation of immune responses (85). This opens new strategies and perspectives for the treatment of inflammatory diseases and autoimmune disorders. For instance, chronic prostatitis type IIIA currently lacks specific treatment methods for this inflammatory condition. However, with the advancement of exosome research, it may become possible to treat this disease by modulating exosome functions (86). Studies indicate that EV play a crucial role in the pathogenesis of chronic prostatitis (87). By regulating the production and secretion of EV, their distribution and functions within the body can be influenced, enabling precise modulation of inflammation. Nonetheless, ensuring the targeted delivery of EV within the body and avoiding their distribution to non-target organs remains a challenge that needs to be addressed (88). Furthermore, the biosafety of EV is a significant concern, necessitating assurance that they do not cause harm to the human body during use.

EV, as an emerging drug delivery system, face numerous challenges but hold significant potential for future applications. With ongoing research and technological advancements, it is reasonable to anticipate that EV will play a more crucial role in the biomedical field, contributing substantially to human health.

## 5 Conclusion/future directions for the field

The diverse biomolecules contained within EV play a crucial role in cellular communication and substance transport. Additionally, they are frequently implicated in research concerning Type IIIA chronic prostatitis (89).

Recent research into the role of EV in the pathogenesis of IIIA chronic prostatitis has yielded the following advancements: EV can contribute to the inflammatory response in chronic prostatitis by carrying and transferring inflammation-related molecules such as interleukins and tumor necrosis factors (90). Studies have shown that, compared to healthy individuals, patients with IIIA chronic prostatitis exhibit significant changes in both the quantity and content of EV in prostatic fluid, suggesting that EV may play a crucial role in the disease mechanism. Furthermore, EV can exacerbate the inflammatory response by binding to receptors on target cells, thereby influencing intracellular signaling pathways (91). Recent research also indicates that EV may be involved in the activation and regulation of immune cells during the progression of IIIA chronic prostatitis.

Based on the aforementioned research advances, EV play a crucial role in the pathogenesis of Type IIIA chronic prostatitis. Consequently, the development of exosome-based therapeutic strategies holds significant potential. Future research could advance in the following directions:

### 5.1 In-depth exploration of extracellular vesicles mechanisms

Further investigation is needed to understand how EV, by carrying and delivering inflammatory molecules, affect the function and physiological processes of target cells. This would help elucidate the pathogenesis of Type IIIA chronic prostatitis and provide a theoretical foundation for developing new therapeutic strategies (92).

### 5.2 Developing new therapeutic strategies based on extracellular vesicles

Utilizing the biological characteristics of EV, new treatment strategies for chronic prostatitis type IIIA can be developed. This includes modulating the production and function of EV to reduce inflammatory responses or designing EV with specific functions, such as drug-loaded EV, to achieve precise disease treatment.

### 5.3 Assessing the potential of extracellular vesicles as biomarkers

Investigating the application value of EV in the diagnosis, monitoring, and prognosis of chronic prostatitis type IIIA can provide new biomarkers for clinical diagnosis and treatment.

### 5.4 Exploring the role of extracellular vesicles in different types of chronic prostatitis

Comparative studies of EV in types of chronic prostatitis other than type IIIA can offer additional insights into the pathogenesis and treatment of chronic prostatitis.

It is worth noting that several challenges currently exist. On one hand, the high cost and complexity of EV isolation and purification present significant issues. Additionally, during detection, cellular debris and other EV in the samples may interfere with the accuracy of identification. On the other hand, the limited drug-loading capacity of EV may result in drug concentrations at target cells or organs being insufficient for therapeutic requirements. Furthermore, the heterogeneity of EV, the absence of standardized identification criteria, technological barriers, and the uncertainty of the regulatory environment are challenges that current EV research must urgently address. However, with ongoing research and continuous technological innovation, it is believed that these difficulties will gradually be overcome, and EV will undoubtedly play an increasingly valuable role in the fields of disease diagnosis and treatment.
